# Biological activities of gastropods secretions: snail and slug slime

**DOI:** 10.1007/s13659-023-00404-0

**Published:** 2023-10-23

**Authors:** Muhammad Rashad, Simone Sampò, Amelia Cataldi, Susi Zara

**Affiliations:** 1 Department of Pharmacy, “G. d’Annunzio” University of Chieti-Pescara, Via Dei Vestini 31, 66100 Chieti, Italy; 2 International Institution of Heliciculture of Cherasco - Lumacheria Italiana Srl, Corso Einaudi 40, 12062 Cherasco, Italy

**Keywords:** Snail slime, Slug slime, Biological activities, Cruelty-free method, Gastropods

## Abstract

Gastropods, a mollusk class including slugs and snails, represent an extraordinarily diverse and ecologically significant group of organisms featuring the largest class of invertebrates. They can be classified as aquatic and terrestrial animals having coiled shells, although some species have reduced or absent shells. Their unique body structure includes a muscular foot for locomotion, a visceral mass containing essential organs, and a distinct head region with sensory organs such as tentacles and eyes. They are used to secrete a complex mixture of glycoproteins, enzymes, peptides, mucus and other bioactive compounds, namely slime, which represents a tool to allow locomotion, protection, and interaction within different habitats. The biological activities of the slime have attracted considerable interest due to their diverse and potentially valuable properties ranging from defense mechanisms to potential therapeutic applications in wound healing, antimicrobial therapy, management of inflammation, and neurological disorders. This review aims at exploring the beneficial effects of snail and slug slime focusing, in particular, on the improvement of the biological processes underlying them. Continued exploration of the intricate components of these slimy secretions promises to discover new bioactive molecules with diverse applications in various scientific and industrial fields.

**Graphical Abstract:**

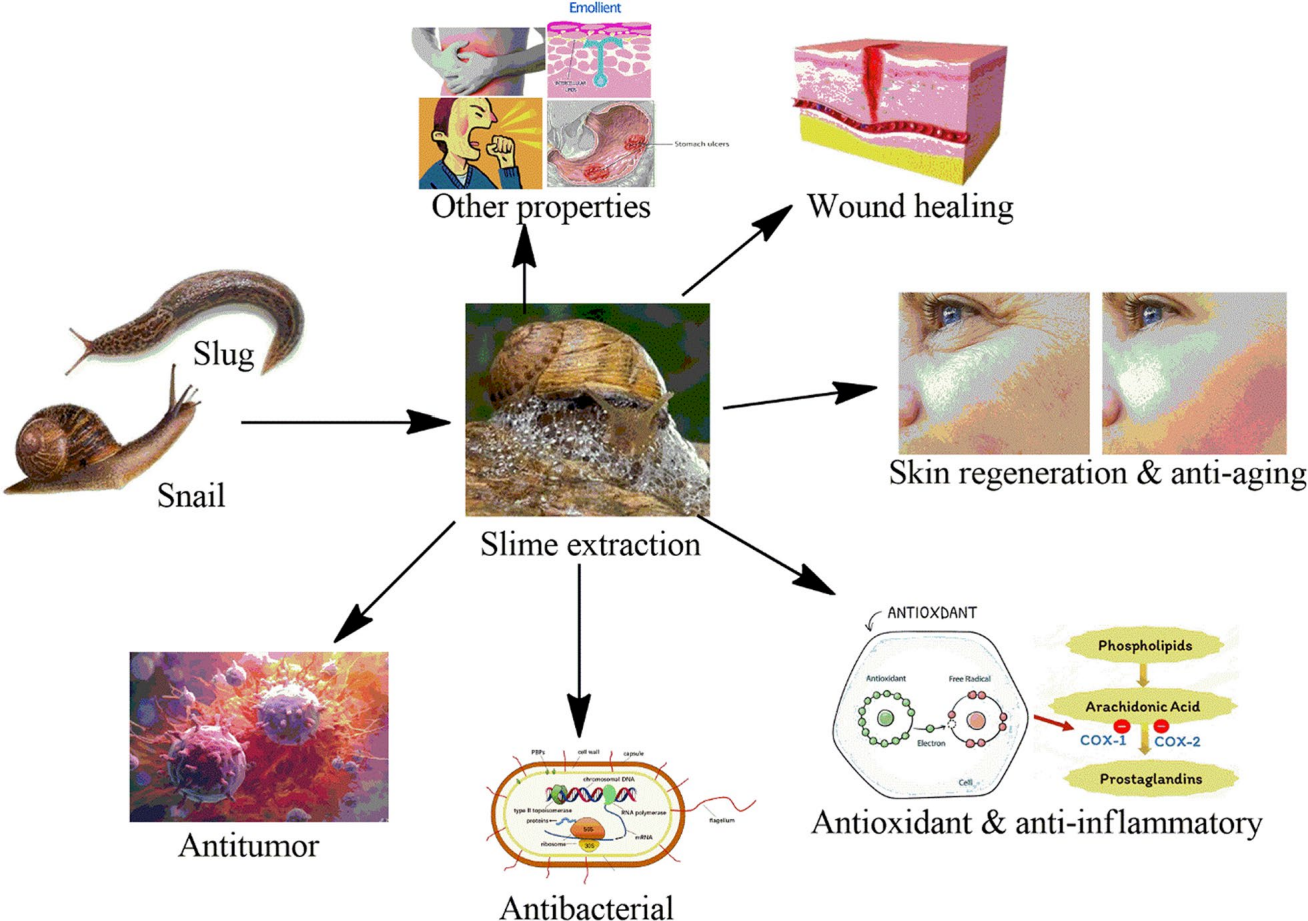

## Introduction

Gastropods, a diverse class of mollusks comprising snails and slugs, have long captivated scientific curiosity owing to their unique adaptations and intriguing biological activities [1]. The most representative biological species of snail include *Helix aspersa,* commonly known as a garden snail, *Helix pomatia*, *Archachatina marginata*, *Achatina fulica*, *Hexaplex trunculus*, *Conus magus*, *Bolinus brandaris* and *Eremina desertorum,* while the most important species of slug is generally represented by *Arion subfuscus* [2, 3].

Among the most distinctive features of these organisms is the production of slime, a mucus-like secretion with a complex composition. The snail and slug slime are watery fluid with 90–99.7% water w/w while the remaining 0.3–10% of slime is known to contain glycoproteins, enzymes, proteoglycans like achacin, hyaluronic acid, glycosaminoglycans, antimicrobial peptides, copper peptides, and metal ions. Snail slime also contains glycolic acid, elastin, collagen, and allantoin; furthermore, the *Helix aspersa* mucus contains Glutathione-S-Transferase (GST) and Superoxide Dismutase (SOD) enzymes. The slug *Arion subfuscus* slime additionally contains a significant amount of metal ions such as copper, iron, manganese, and zinc which are essential for gel formation upon slime secretion [3].

Snail and slug secretions play a pivotal role in the survival and interaction of the animals with the environment. The study of gastropod slime has gained substantial attention in recent years due to its multifaceted properties, including its defensive mechanisms, wound healing potential, antimicrobial activities and applications in skincare and cosmetics [4].

Gastropods inhabit a wide range of ecosystems, from terrestrial landscapes to freshwater bodies and marine environments. Their slime, a complex concoction of several bioactive compounds, serves as a versatile tool that contributes to their locomotion, protection, and interaction within these diverse habitats [5, 6].

When faced with threats, snails and slugs can release copious amounts of slime that create a slippery and adhesive barrier, deterring predators and making escape more feasible [7]. Additionally, certain species incorporate chemical deterrents into their slime, an adaptation that highlights the remarkable interplay between chemical and physical defenses in their survival strategies.

Beyond defense, gastropod slime exhibits further beneficial properties. In fact, considering that it has been demonstrated that the components found within the slime appear able to expedite essential cellular processes, some potential applications in medical contexts, such as wound healing and tissue regeneration, can be also hypothesized [8]. Furthermore, the presence of antimicrobial peptides and proteins within the slime opens avenues for novel antimicrobial agents, particularly relevant in the current era of escalating antibiotic resistance [9].

The cosmetic and skincare industry has also seized upon the potential benefits of gastropod slime. Compounds like hyaluronic acid and glycoproteins found in the slime are believed to have moisturizing and skin-smoothing effects, resulting in the integration of slime extracts into various beauty products [10].

Additionally, the neurological implications of certain slime components, including neuroprotective properties and potential contributions to neurodegeneration, represent a promising avenue for research in neurological disorders and therapeutic strategies [11].

As we embark on a journey into the intricate world of gastropod slime, this review seeks to uncover the biological activities underpinning its multifarious functions. By exploring the mechanisms behind slime production, its ecological significance, and above all its potential applications, purpose of present review is to deepen the knowledge of snail and slug slime biological activity in processes such as wound healing, aging, inflammation, tumor, and some bacterial diseases along with the Muller strategy to extract the mucus. Through comprehensive examination and analysis, this review provides knowledge surrounding gastropods' snail and slug slime, with implications ranging from ecological interactions to innovative biomedical and cosmetic advancements.

## Biological activities of snail and slug slime

### Wound healing properties

Wound care continues to pose significant challenges in clinical settings due to the prevalent occurrence of traumatic injuries and persistent chronic wounds [12]. Conventional approaches involving surgical sutures and staples are established norms for reconnecting damaged tissues and sealing wounds. However, these methods are associated with potential drawbacks including discomfort, the risk of surgical site infections, and the likelihood of resulting skin scarring [13]. To address these concerns, alternative treatments have emerged, among them tissue adhesives, like fibrin glue, and adhesives based on polyethylene glycol, are being used. These alternatives exhibit a higher level of effectiveness and being also less invasive, cause less pain [14]. In recent years, progress in understanding natural adhesion phenomena and underlying molecular and biological mechanisms has paved the way in order to create a new generation of tissue adhesives [15]. This advancement holds promise for revolutionizing wound closure techniques and improving patient outcomes.

Snail and slug mucus have gained attention for their potential wound healing properties and the scientific knowledge in this area is still evolving [2]. While some studies suggest positive effects, more research is required to completely comprehend the mechanisms and establish the effectiveness of these substances for wound healing. The mucus generated by the slug species *Arion subfuscus* comprises a combination of anionic polymers and positively charged proteins that are intricately intertwined. This slug mucus lacks adhesiveness for human skin; to overcome this problem proteins from the mucus were extracted and mixed with commercially available polymers to promote the adhesion and wound healing potential [16]. This double network hydrogel proves an effective treatment of wound healing in a dose dependent-manner and the optimal protein concentration found was 1–1.5 mg/mL. Moreover, it has been recognized that the snail slime obtained from the *Helix* species like *Helix pomatia* is able, on one hand, to significantly increase the growth of fibroblasts by activating the Erk mechanism, to promote the angiopoietin-1 gene expression and, on the other hand, to appreciably reduce cytotoxicity after 24 and 48 h of treatment [8]. Additionally, slime obtained from the *Helix aspersa* has shown a good capacity to stimulate skin regeneration after acute radiodermatitis, a major side-effect of radiotherapy [17, 18]. The wound dressings with the mucus secretions of *Achatina fulica* boosted the maturation of granulation tissue and the collagen deposition rate, which speed up the healing process [2, 19]. Ziconotide (SNXIII), a synthetic peptide obtained from sea snail *Conus magus* venom, which has been under FDA review since 1999, is a distinct remedy to cure pain caused by abscesses, burns, and related wounds. This venom or slime includes conotoxins peptides which possess a strong neurotoxic effect, 1000 times more potent than morphine, and that are used to treat pain by blocking calcium channels [20]. Wound healing with snail slime can be one valid alternative approach to the standard ones as it is not only easy to use, but also capable to efficiently spread through the skin. In addition, it does not clog skin pores, and possesses an antibacterial effect (see Antibacterial activities paragraph) [21].

### Skin regeneration and anti-aging properties

Snails offer a huge array of bioactive compounds that hold potential for applications in both the cosmetic and pharmaceutical sectors. These compounds can serve as valuable resources for the creation of novel formulations with reduced toxicity and after-effects, as compared to conventional compounds commonly employed in these industries [22]. Bioactive components sourced from various snail-derived materials, including crude extracts, mucus, and slime, encompass a range of molecules such as glycans, polypeptides, and proteins [23]. These elements present opportunities for addressing a spectrum of health concerns, including viral lesions, warts, and various dermatological issues [24]. The mucus of *Cryptomphalus aspersa* mollusk promotes skin regeneration by inducing fibroblasts proliferation and maintaining the actin of cytoskeleton, regulates the metalloproteinase activity and activates extracellular matrix assembly and promote skin regeneration through a molecular mechanism associated with basic Fibroblast Growth Factor (bFGF) [25].

**Fig. 1:**
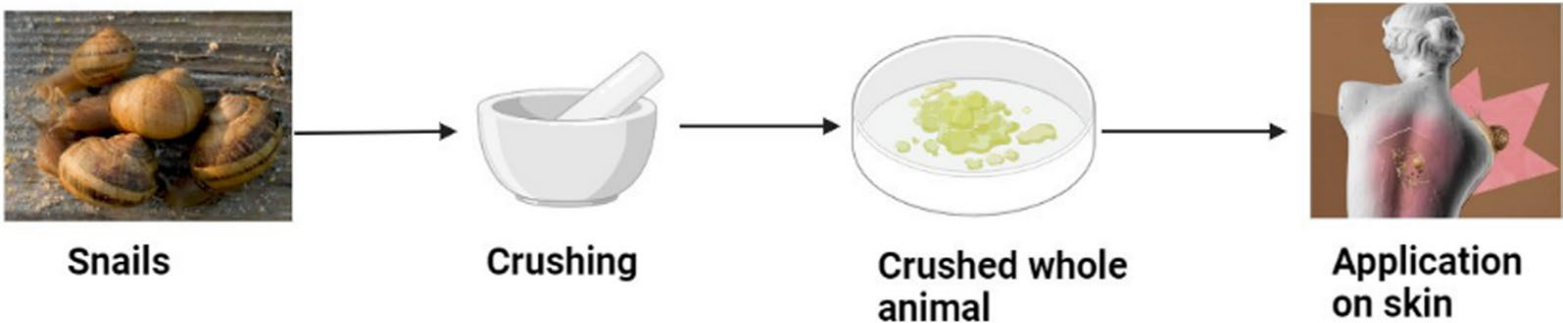
Application of crushed whole snail to treat skin disorders

In ancient Greece, people possessed traditions to treat inflamed skin and other skin disorders, such as warts, with crushed snails finding that it appeared useful to cure those skin disorders (Fig. 1) [26]. Similarly, the slime obtained from the *Helix aspersa* has been demonstrated to significantly increase the fibroblast viability by positively regulating the cell cycle and elevating the collagen 1 secretion [8].

Mucin proteins, included in snail mucus, can be used for several biological functions, including adhesion, lubrication, and microbial protection [2]. Mucin obtained from *Helix aspersa* has shown a significant reduction of melanin and tyrosinase activity on murine melanoma B16F10 cell line (IC_50_ value of 288 μg/mL and 286 μg/mL, respectively), without affecting cell viability [27]. In 2010, a snail cream, Aqua Cell Renew, claimed that 70% snail extract effectively promotes skin regeneration and skin healing.

Products with snail slime are hailed as the next wonder face-fixer in the United States, where they are used to cure acne, to decrease pigmentation and scarring, and to counteract wrinkles [2]. The secretions of *Achatina fulica* and *Cornu aspersum* are frequently featured in several revolutionary skincare products due to their favorable dermatologic qualities associated with the antioxidative effect of bioactive glycosaminoglycans [28]. Laneri et al*.* [29] claimed that the glycolic acid and allantoin contents present in the *Helix aspersa* mucus were very effective in skin regeneration and anti-aging; Liu et al*.* claimed that the recent craze implies the use of snail slime to reduce wrinkles, scarring, and pigmentation [30].

In conclusion, snails and slug slime have gained attention in the skincare industry for their potential anti-aging and skin care benefits. These natural substances contain a variety of compounds that are believed to contribute to healthier and more young-looking skin. Snail and slug slimes are rich in hyaluronic acid, a natural compound that helps retain moisture in the skin [31]. Keeping the skin hydrated is vital for maintaining elasticity and minimizing the visibility of lines and wrinkles. The slime also contains glycoproteins and peptides that have the potential to stimulate the production of collagen and elastin [32] thus contributing to the maintenance of the firmness and elasticity of skin. Additionally, certain bioactive molecules present in the slime including growth factors may aid in skin regeneration by facilitating cell turnover and replacing dead cells with healthy ones [33]. Multiple effects of snail slime help to improve skin hydration, increase collagen production, and enhance skin regeneration, which could potentially result in a reduction of wrinkles and aging. Moreover, some specific compounds found in snail and slug slime, like allantoin, are believed to possess wound healing properties that might help minimize the visibility of scars [34].

### Antioxidant and anti-inflammatory properties

**Fig. 2:**
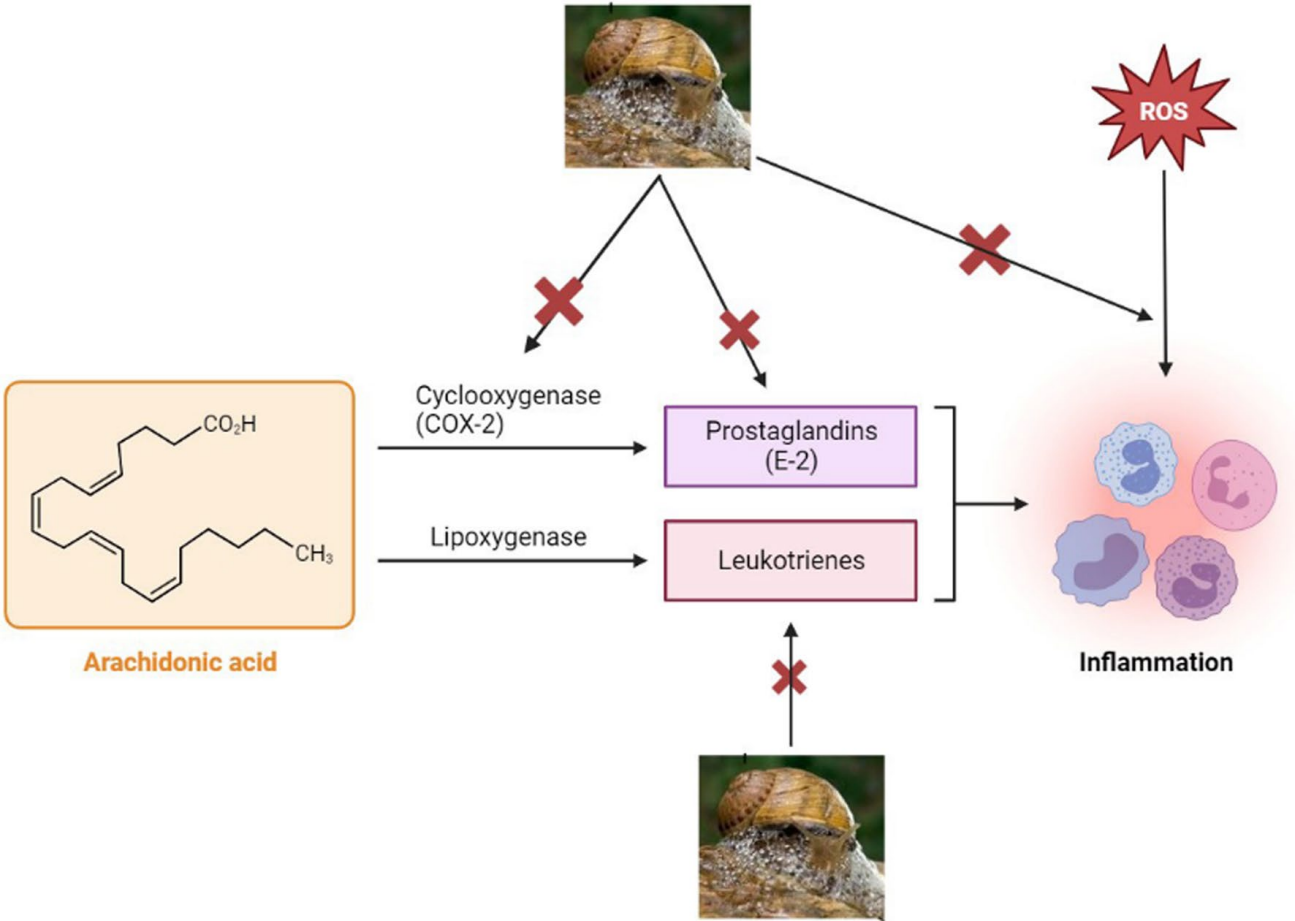
Mechanism of snail slime effect in the inhibition of inflammation

The curiosity in the snail and slug mucus trails dates back to ancient Greece, where people were used to apply the mucus for its potential to decrease inflammation and to act as an antioxidant [35]. As already above mentioned, Greeks traditionally used crushed snails to treat skin inflammation [26], on the other hand, in Italy, rubbing the mucus of a common garden slug over the skin helped heal acne, warts, dermatitis, calluses, and inflammations [36]. Additionally, mucins have demonstrated the capacity to cure internal wounds in addition to treating surface lesions. Mucins have been used with nonsteroidal anti-inflammatory drugs (NSAIDs) in order to decrease or eradicate stomach mucosal damage [37]. A study also showed that slime significantly reduced the immunological response elicited by IgG and IgM antibodies, as well as the activation of fibrinogen, a recognized inflammatory factor [2, 38, 39]. Garden snail slime was found to alleviate erythema and accelerate healing in rat models by regulating antioxidants and free radicals [40, 41]. Rizzi et al*.* confirmed the *Helix aspersa* Muller’ slime antioxidant activity by using the 2,2-diphenyl-1-picrylhydrazyl (DPPH) and 2,2'-Azinobis (3-ethylbenzothiazoline-6-sulfonic acid)-diammonium salt (ABTS) methods and the anti-inflammatory effect by measuring the tyrosinase inhibition activity [4]. Ahmad et al*.* validated all the listed in vitro, in vivo, and human clinical trials that have claimed the anti-inflammatory and antioxidant activities of molluscan natural products finding a potential outcome [42]. Ricci et al*.* evidenced that the snail slime reduces the inflammatory events by inhibiting the cyclooxygenase-2 (COX-2) gene expression in human gingival fibroblasts exposed to an inflammatory stimulus represented by hydrogen peroxide (H_2_O_2_) [8]. Tsvetanova et al*.* investigated the effect of *Helix aspersa* slime on scopolamine-induced cognitive impairment and oxidative stress in rat brain [11]. The authors claimed that an oral administration of snail slime (0.5 mL/100 g) for 11 days helps to regulate the oxidative stress and hypothesized that it can be used to treat impaired cognitive functions as it is could be able to reduce neurodegenerative process by inhibiting the endogenous cellular stress. Ibrahim et al*.* investigated the anti-inflammatory and antioxidant properties of *Eremina desertorum* mucus on carbon tetrachloride (CCl_4_) induced intestinal inflammation and tested damage on male albino mice [43]. They found that treatment with snail mucus restored all the antioxidant and anti-inflammatory parameters (decreased the caspase-3, interleukin-2, C-reactive protein, and lipid peroxidation while increased the catalase activity, total proteins levels, glutathione reductase, SOD, 17β estradiol and testosterone levels), as showed in Fig. 2. Research on freshwater snail extract and seawater gastropod extract on induced paw edema rats showed that they have good anti-inflammatory effect as they are able to lower the concentration of tumor necrosis factor-alpha (TNF-α) (pro-inflammatory cytokine) and increase interleukin-10 (anti-inflammatory cytokine) [44], the antioxidant activity was also confirmed by DPPH and ABTS methods. Mucus extracts from *H. aspersa* and *E. desertorum* were tested for their anti-inflammatory activity and compared to the standard aspirin effects: results showed the membrane stabilization of human red blood cells, proteinase inhibitory and albumin denaturation activity [45]. In conclusion, snail and slug mucus beneficial effect in the treatment of inflammatory events has been widely demonstrated and they can be thought as alternative products to promote and/or enhance the free radicals scavenge.

### Antibacterial activities

Antibiotic-resistant bacteria are growing to an alarming rate with limited available resources. Effective remedies have been produced by research on naturally occurring antibacterial compounds. Snails and slugs’ feet are a prominent source of contamination and get infected by different bacterial or viral diseases. They produce and secrete the mucus which can kill the bacteria and protect the animals from infection [2]. Different studies concluded that this mucus can promote antibacterial activity. *Helix aspersa* slime had a strong antibacterial activity against several strains of *Pseudomonas aeruginosa* and less effective against *Staphylococcus aureus*, mucus from *Achatina fulica* also inhibited the growth of *S. aureus* [46], *Achatina fulica*’s mucus has been reported to contain two proteins, achasin and mytimacin, with broad-spectrum antibacterial activity against the *Staphylococcus aureus*, *Bacillus subtilis*, Gram-negative bacteria, Gram-positive bacteria, *Pseudomonas aeruginosa* and *Escherichia coli* [2, 47]. *Achatina fulica*’s mucus also inhibited the bacterial growth of both *Staphylococcus epidermidis* and *Staphylococcus aureus* when administered to a mouse model through wound dressing films [48]. *Helix aspersa* mucus also showed antimicrobial activity against several strains of *Pseudomonas aeruginosa* [46, 49]. Slime of both *Archachatina marginata* and *Achatina fulica* were administered as wound dressing on 28 clinical wound samples, collected with known common infections, and confirmed the anti-bacterial potency against *Pseudomonas, Streptococcus* and *Staphylococcus* isolated from wounds [50–52]. El-Zawawy and Mona [45] investigated that mucus extract of *Eremina desertorum* was the most effective against the resistant strains *Staphylococcus aureus* and *Escherichia coli* with a strong inhibitory activity. Lopez et al*.* also demonstrated that crude extracts of marine snail *Cenchritis muricatus*, inhibited the development of *Staphylococcus aureus* and showed an *Escherichia coli* growth inhibition of 95.9% [53].

### Anti-tumoral activities

Snail mucus has shown therapeutic effects on melanoma, one of the most serious skin tumors. *Helix aspersa* slime showed significant inhibition of cell viability after 72 h of treatment using 300 µg/mL slime on IGR-39 and SK-MEL-28 (melanoma cell lines), due to an apoptotic effect associated with Poly (ADP-ribose) polymerase (PARP) cleavage [27]. Furthermore, metastasis was inhibited by limiting integrin activity and expression, consequently counteracting the cancer progression [54]. In another study, *Helix aspersa* slime inhibited the growth of IGR-39 and SK-MEL-28 melanoma cells, by increasing expression of the cytokine Tumor Necrosis Factor (TNF-α), and inhibiting the transcription process, by blocking transcription Nuclear factor kappa light chain enhancer of activated B cells (NF-κB), that in proper regulation has been linked to cancer progression [55]. An in vitro study revealed that *Helix aspersa* slime had an anti-tumoral effect against human melanoma cells in addition to treating melanogenesis. [56]. Atta et al*.* confirmed the anti-tumoral activities of *Eremina desertorum* snails´ mucus against human colon adenocarcinoma (CACO-2) and human hepatoma (HepG-2) cell lines [57]. *Hexaplex trunculus* and *Bolinus brandaris* are sea snails, their slime reduced glioblastoma cells adhesion, moreover, U87 cells treated with *Hexaplex trunculus* and *Bolinus brandaris* mucus significantly decreased their proliferation rate by 50% and 70%, respectively [58]. Chien et al*.* [59] demonstrated that snail mucus significantly reduced the viability of triple-negative MDA-MB-231 breast cancer cell line with considerably reduced cytotoxicity to normal breast epithelial cells and increased their sensitivity to chemotherapy through activation of Fas signaling by nucleolin inhibition.

### Other biological activities

Apart from the specific biological activities explained above, some researchers also investigated other beneficial effects of snail slime. For example, snail mucus has also been used to treat nose bleeding, stomach upsets, and burns-related pain [20]. Gastropod secretion was also positively linked to fertility and femininity [60] and were considered to hasten delivery and combat female scrofula due to its emollient effect. It had been used to treat gastritis or stomach ulcers due to its emollient, anti-inflammatory and antibacterial effects [61]. *Helix pomatia* mucus inhibits the prostaglandin-E2 activities and also possesses emollient and antibacterial properties, due to which it was considered an attractive remedy for whooping cough and chronic bronchitis such as tuberculosis [62]. Snail mucus have strong antioxidant effects and it is hypothesized that it can be also used to treat impaired cognitive functions of brain and to improve cardiovascular protection [63].

## Snail and slug slime extraction methods

Natural products provide a key role in the discovery of many useful drugs against deadly diseases. They can be easily obtained by a variety of cost-effective methods. Natural products, obtained from plants or animals, sometimes lead to their death and/or an injury to the source. Snails and slugs’ mucus had been used for a long time ago, in ancient Greece and China, people have been using these small animals for their beneficial effects against multiple skin disorders; people used to crush the whole animal and apply it onto the skin. These old techniques allowed people to treat skin disorders although the animal life had not been preserved thus causing significant damage to mollusks survival. Afterward, in previous but recent researches, the extraction of snail slime still led to the killing of the animals. Wiya et al. extracted the snail slime by placing the snails on a plate in warm (32 °C) water and then scrubbing the outer shell and slime was squeezed from the animal, leading to animal death [64]. Similarly, Onyema and Adikwu, both extracted the snail slime by mechanically removing the outer shell and then scrubbing the fleshy animal, leading to their death [65, 66].

**Fig. 3:**
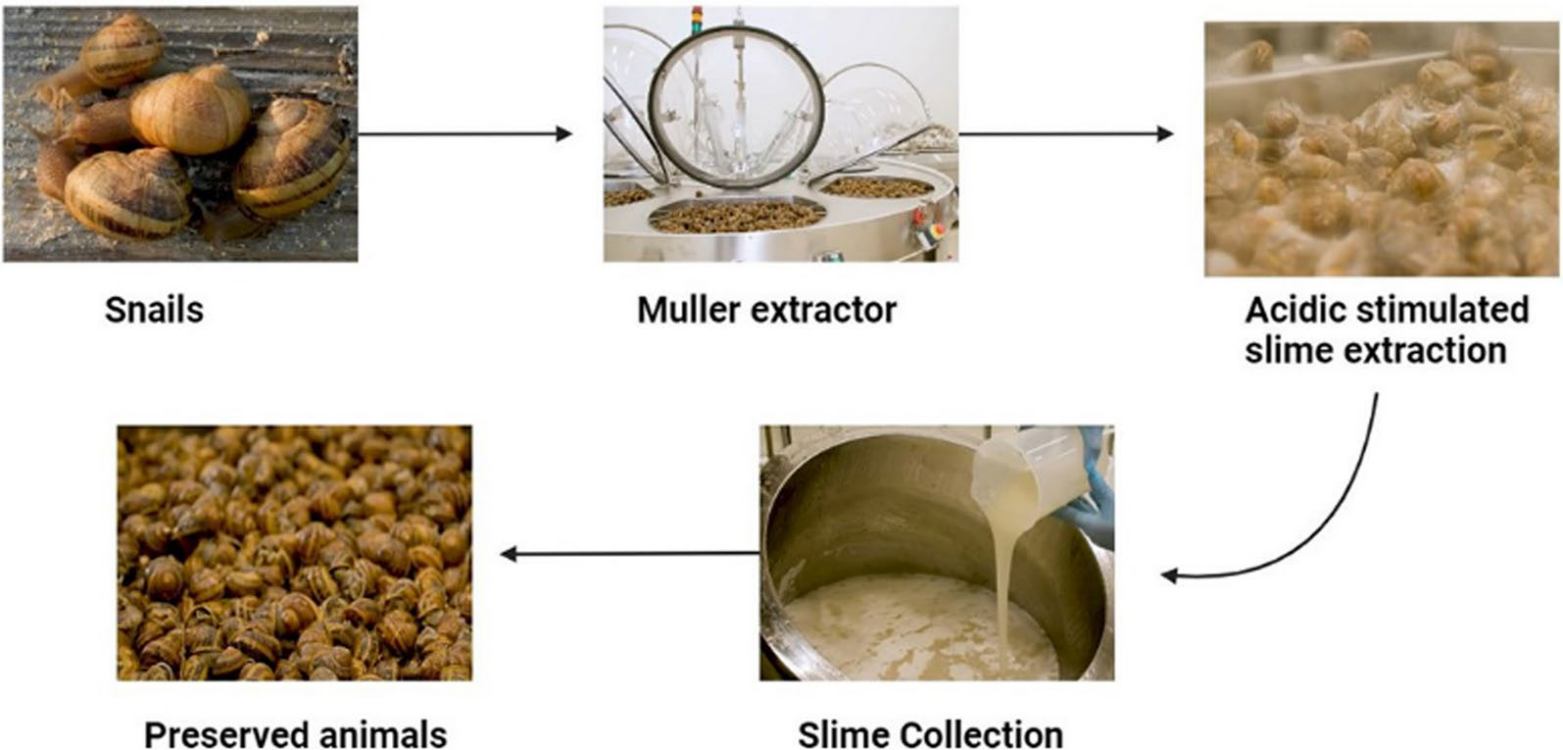
Depiction of Muller extraction, a cruelty-free process (figures obtained from Lumacheria Italiana srl)

Nowadays, animal ethics focus on the minimal use of animals for research purposes and to save their lives [67]. In this light, the innovative and high yield-producing Muller extractor (Lumacheria Italiana srl, Cherasco, Italy) (Fig. 3) is used for multiple extractions of slime preserving animals’ life with highly cost effectiveness. The Muller method in fact, implies that the harvested adult snails, 12–15 months old, are first cleaned of dead and/or injured/damaged animals, then washed with running water to remove dust or any other foreign particles. After that, snails are then subjected to Muller extractor where they are first sprayed with ozonized water intermittently for 30 min to remove pathogens and contaminating agents and to awaken the animals. Afterwards, snails are sprayed again for 30 min with an acidic solution to stimulate stress-free slime secretion allowing animals survival [8].

## Conclusions

It is widely believed that natural products were the source of the majority of active pharmaceutical components used in medicine development. More than 80% of all pharmaceutical drugs were natural or produced from natural sources. Side and/or adverse effects of synthetic products have encouraged the availability and usage of natural products especially for skin care and cosmetic products. Natural products have several advantages respect to synthetic ones: they are easily available, generally they can be obtained in a very cost-effective way, and they require minimal use of resources. Snails and slugs are a precious source of viscous secretions slime which is attracting attention for its promising characteristics. Many studies investigated and confirmed the beneficial effects of gastropods slime, including the antibacterial, anti-inflammatory and antioxidant, anti-tumoral, anti-aging, tissue regeneration, wound healing, and many other properties. It has been used in Italy since ancient times for beauty products, and to cure skin related disorders. Moreover, innovative extraction methods, e. g. Muller’s method, preserving the animal life, opens the possibility to obtain a high yield of product multiple times from a single animal and above all avoiding dangerous perturbation in the mollusks ecosystem. Further investigation is required to explore the beneficial effects of this precious product for the betterment of human health.

## Data Availability

Not available.
